# SonarReg-GS SLAM: Sparse Sonar-Guided Depth Regularization for Underwater Gaussian Splatting SLAM

**DOI:** 10.3390/s26154713

**Published:** 2026-07-24

**Authors:** Wen Yang, Xiaolong Qian, Xulin Liu, Jianxing Leng

**Affiliations:** 1 Ocean College, Zhejiang University, Zhoushan 316021, China; wenyang@zju.edu.cn (W.Y.); 12134090@zju.edu.cn (X.L.); 2 College of Optical Science and Engineering, Zhejiang University, Hangzhou 310027, China; xiaolongqian@zju.edu.cn

**Keywords:** underwater SLAM, 3D Gaussian Splatting, forward-looking sonar, visual–acoustic fusion

## Abstract

3D Gaussian Splatting (3DGS) SLAM provides an explicit scene representation for dense tracking and mapping, which is useful for underwater robotic perception. However, underwater monocular 3DGS SLAM lacks reliable metric depth cues: monocular depth estimation can provide dense structural priors, but its scale and reliability often degrade under underwater appearance changes. Forward-looking sonar (FLS) provides range–azimuth acoustic measurements whose range coordinate is related to physical distance, but raw sonar observations are sparse, noisy, and ambiguous. Our key insight is that FLS returns can serve as sparse metric depth anchors when they are associated with visually detected object regions. Based on this insight, we propose SonarReg-GS SLAM, an underwater visual–acoustic 3DGS SLAM framework with sparse sonar-guided depth regularization. Given synchronized RGB and sonar inputs, SonarReg-GS SLAM uses object masks to constrain the search space for acoustic range association. Filtered sonar responses are selected as sparse metric anchors through object-aware sampling, bearing-to-beam gating, and valid-pair checking. These anchors regularize the scale of monocular depth and generate metric depth priors for Gaussian initialization and tracking. An object-aware RGB mask loss further increases supervision on detected object regions while preserving full-scene mapping. Experiments on two public RGB–sonar underwater datasets show that SonarReg-GS SLAM improves tracking accuracy and mapping quality compared with representative classical SLAM and Gaussian Splatting SLAM baselines. Compared with Splat-SLAM, our method reduces the average ATE RMSE from 0.1296 m to 0.1015 m on UXO and from 0.5687 m to 0.4640 m on OPTI, corresponding to relative reductions of 21.7% and 18.4%, respectively. For rendering-based mapping, it increases the average PSNR from 28.05 dB to 29.61 dB on UXO and from 20.91 dB to 28.63 dB on OPTI while reducing the average LPIPS from 0.345 to 0.173 and from 0.450 to 0.303, respectively.

## 1. Introduction

Autonomous underwater vehicles (AUVs) and remotely operated vehicles (ROVs) are widely used in subsea inspection, marine surveying, intervention, and object search tasks such as unexploded ordnance (UXO) localization [1,2]. These tasks require simultaneous localization and mapping (SLAM) to estimate robot motion and build metrically consistent maps of the surrounding environment [3,4]. However, underwater visual perception differs from terrestrial imaging because of light attenuation, wavelength-dependent color shift, scattering, caustics, and reflections [5,6]. Commodity active RGB-D sensors are also often limited underwater due to attenuation, scattering, and refraction [7]. Therefore, many underwater SLAM systems still rely on monocular or vision-dominant inputs, which are affected by scale ambiguity, scale drift, and underwater appearance changes [3,4]. Forward-looking sonar (FLS) provides acoustic range measurements that do not depend on visual texture and are related to physical distance [8,9]. This makes sonar useful for providing metric constraints when monocular vision lacks absolute scale [10,11]. However, raw FLS observations are sparse, ambiguous, and noisy. Limited angular resolution, elevation ambiguity, multipath reflections, and reverberation can produce incorrect or duplicated returns, especially around reflective objects, metallic targets, and confined environments such as pools or tanks [11,12]. Thus, raw sonar measurements cannot be directly treated as dense and unambiguous geometric observations. In this work, we use sonar to provide sparse metric cues for visual geometry regularization.

Recently, 3D Gaussian Splatting (3DGS) has provided explicit scene representations for efficient differentiable rendering [13]. Its rendering efficiency has further motivated Gaussian-based SLAM systems for dense tracking and mapping [14,15]. Applying 3DGS SLAM underwater remains challenging because Gaussian mapping depends on geometric initialization. Depth errors affect where Gaussian primitives are inserted, how their spatial scales are initialized, and how later optimization proceeds. Although monocular depth foundation models can provide dense depth predictions [16,17,18], underwater images may differ from their training data because of color shift, caustics, reflections, low-texture surfaces, and underwater-specific object appearances. These depth errors can lead to inaccurate Gaussian placement and scale initialization during incremental reconstruction. For underwater scenes, 3DGS-based methods have incorporated underwater image formation models, attenuation–backscatter modeling, or uncertainty-aware visual geometry into reconstruction and SLAM [6,7,19,20]. Sonar-aware Gaussian methods have also shown that acoustic measurements can be useful for underwater reconstruction and sonar view synthesis [21,22,23].

However, existing underwater 3DGS SLAM systems mainly rely on visual inputs, where geometric priors are usually obtained from monocular depth estimation, visual tracking, or tracking-derived uncertainty. In contrast, sonar–Gaussian methods mainly focus on reconstruction or acoustic rendering rather than visual SLAM. How to use sparse and noisy sonar measurements as metric constraints for 3DGS SLAM remains insufficiently explored.

To address this problem, we propose SonarReg-GS SLAM, an object-aware visual–acoustic underwater 3D Gaussian Splatting SLAM framework with sparse sonar-guided depth regularization. The key idea is to use sonar as a sparse metric regulator for monocular depth rather than directly inserting raw sonar measurements into the Gaussian optimizer. Given an RGB image, we first extract object masks using open-vocabulary segmentation models [24]. These masks are not used for dense semantic mapping. Instead, they provide weak spatial priors to constrain acoustic range extraction. Inspired by object-level opti-acoustic range association [11], sampled mask rays are matched to corresponding sonar beams, where thresholded dominant echo responses are selected as sparse sonar anchors after target-class filtering, near-contour sampling, bearing-to-beam gating, and valid-pair checking.

The extracted sonar anchors provide sparse range constraints with physical scale, while monocular depth provides dense structural information. We use these anchors to estimate and regularize the scale of monocular depth, producing dense metric depth priors for Gaussian initialization and tracking. Our system reconstructs the entire scene as a dense Gaussian map while using object masks to enhance detected object regions during optimization. Specifically, the object-aware loss keeps the standard full-image photometric and depth supervision and assigns higher weights to pixels inside the detected object masks. In this way, the system still reconstructs the full scene while improving local geometry and appearance in object regions.

In contrast to existing visual–acoustic SLAM systems that typically use sonar observations as sparse landmarks, range factors, or pose constraints, SonarReg-GS SLAM converts filtered, object-conditioned FLS returns into sparse metric depth anchors for monocular depth regularization. Compared with monocular 3DGS-SLAM methods, SonarReg-GS SLAM retains the same objective of camera tracking and dense full-scene Gaussian mapping but introduces external acoustic range cues to regularize the monocular depth prior before it is used in the subsequent SLAM pipeline. The resulting sonar-regularized depth is used for dense tracking, Gaussian initialization, and depth supervision during mapping. Unlike sonar-guided Gaussian reconstruction or novel view synthesis methods, SonarReg-GS SLAM focuses on RGB–FLS tracking and dense Gaussian mapping, with sonar serving as an external geometric prior rather than as a dense scene representation or acoustic rendering signal.

We evaluate the proposed SonarReg-GS SLAM on two public RGB–sonar underwater datasets: an object-centric UXO dataset [12] and a pool-based OPTI dataset [11]. Compared with representative classical SLAM and 3DGS SLAM baselines, our method shows improved tracking accuracy and mapping quality, indicating that the introduced sonar modality contributes useful metric constraints for underwater Gaussian Splatting SLAM.

The main contributions of this work are summarized as follows:

- We present SonarReg-GS SLAM, an underwater visual–acoustic 3D Gaussian Splatting SLAM framework that integrates synchronized RGB and forward-looking sonar inputs for tracking and dense full-scene Gaussian mapping.
- We introduce a sparse sonar-guided depth regularization mechanism that uses filtered FLS range anchors to generate metric depth priors for Gaussian initialization and tracking.
- We design an object-aware RGB mask loss that increases photometric supervision in detected object regions without restricting the system to object-only mapping.

The remainder of this paper is organized as follows. Section 2 reviews related work on underwater visual–acoustic SLAM, Gaussian Splatting-based SLAM, and sonar-guided reconstruction. Section 3 introduces the preliminaries of 3D Gaussian Splatting and forward-looking sonar imaging geometry. Section 4 presents the proposed SonarReg-GS SLAM framework, including sonar-guided depth regularization, dense tracking, and Gaussian mapping with object-aware supervision. Section 5 describes the experimental setup and presents the quantitative comparisons, qualitative results, ablation studies, and robustness analyses. Section 6 discusses the limitations of the proposed method and possible directions for future work. Finally, Section 7 concludes the paper.

## 2. Related Work

### 2.1. Underwater Opti-Acoustic SLAM

Underwater SLAM is commonly studied under strong sensing constraints, since GPS is unavailable and visual measurements are affected by medium-induced appearance changes. Surveys on underwater visual SLAM have summarized these challenges and the limitations of vision-only systems [3,25]. Cameras provide rich texture and semantic information, but monocular systems lack absolute scale. Forward-looking sonar (FLS) complements cameras by providing range–azimuth acoustic measurements that do not rely on visual texture and are less sensitive to illumination changes, as discussed in optical–acoustic sensing studies [26]. However, FLS observations are sparse and affected by limited angular resolution, elevation ambiguity, multipath reflections, and reverberation [12]. These complementary properties have motivated visual–acoustic SLAM systems that combine cameras, sonar, inertial sensors, DVL, and depth measurements. SVIn2 [4], for example, integrates sonar, visual, inertial, and depth measurements for underwater state estimation.

A related line of work studies cross-modal visual–acoustic association. Direct feature matching between optical and acoustic images is difficult because the two modalities follow different image formation processes. The opti-acoustic epipolar geometry studied by Negahdaripour [8] provides an early geometric basis for camera–sonar association. Recent object-level visual–acoustic methods reduce cross-modal ambiguity by using image segmentation to guide the association between camera observations and sonar ranges. Opti-Acoustic Semantic SLAM [11], for instance, associates image object masks with acoustic range returns to estimate object landmarks in a semantic SLAM factor graph. Existing visual–acoustic SLAM methods typically use sonar measurements as sparse landmark or range constraints for pose optimization. In contrast, our sonar-anchor extraction adopts a related object-conditioned association principle but uses it for a different purpose: selecting filtered metric anchors to regularize monocular depth before Gaussian initialization and tracking.

### 2.2. Gaussian Splatting for Dense SLAM and Underwater Mapping

Classical feature-based SLAM systems provide efficient tracking but usually reconstruct sparse maps. ORB-SLAM3 [27] is a representative example of this line of work. Neural implicit SLAM methods, including iMAP [28] and NICE-SLAM [29], enable denser scene representations, but they rely on volumetric sampling and repeated network queries based on NeRF-style implicit representations [30]. In contrast, 3D Gaussian splatting represents scenes with explicit Gaussian primitives and supports efficient differentiable rasterization [13]. Recent Gaussian SLAM systems, such as MonoGS [14], Splat-SLAM [31] and SplaTAM [15], adopt this representation for dense tracking and mapping.

Underwater scenes introduce additional challenges because standard Gaussian rendering assumes a clear medium. SeaSplat [19] and WaterSplatting [20] incorporate underwater image formation models, attenuation-backscatter modeling, or hybrid medium representations to improve underwater reconstruction and rendering. More recent underwater 3DGS SLAM systems further extend Gaussian representations to tracking and mapping. UWGS-SLAM [7] introduces a learnable medium model into monocular underwater Gaussian SLAM, while DUV-SLAM [6] propagates tracking-derived depth uncertainty into medium-aware Gaussian mapping. These methods improve underwater Gaussian SLAM from a vision-centric perspective. However, their geometric priors are still derived from monocular depth estimation, visual tracking, or tracking uncertainty, without using FLS measurements as external metric anchors for depth regularization.

### 2.3. Sonar-Guided Reconstruction and Object-Conditioned Association

Several recent studies have explored sonar-guided reconstruction and sonar-aware Gaussian representations. SonarSweep [32] estimates depth by warping sonar features into the camera reference frame through a plane-sweeping formulation. Z-Splat [21] introduces a Gaussian splatting formulation for camera–sonar fusion and improves depth-axis reconstruction using sonar measurements. Aqua-Splat [33] investigates sonar–camera Gaussian reconstruction, while SonarSplat [23] focuses on imaging–sonar view synthesis. These works show that acoustic measurements can provide useful geometric cues for underwater reconstruction and rendering. However, they mainly focus on reconstruction or novel-view synthesis rather than underwater SLAM.

A key issue associated with the use of sonar in visual SLAM is how to select reliable acoustic cues from sparse and ambiguous sonar observations. Instead of directly treating raw sonar returns as dense geometric inputs, visual priors can be used to restrict the acoustic search space. Object masks provide a practical form of such priors, since they indicate image regions that are more likely to correspond to relevant sonar responses. Foundation models such as Segment Anything [34] and Grounded-SAM [24] can provide these masks without dense task-specific annotations. In our system, the masks are used as weak spatial priors rather than dense semantic labels. They constrain the sonar-anchor search space and reweight detected object regions during Gaussian optimization. Unlike sonar-aware Gaussian methods that mainly use acoustic observations for reconstruction or rendering, our method extracts filtered sonar anchors from object-conditioned image regions and uses them to regularize monocular depth before Gaussian initialization.

In summary, SonarReg-GS SLAM differs from related methods mainly in the role assigned to sonar and the system objective. Existing visual–acoustic SLAM systems [4,11] typically use sonar measurements as sparse landmarks, range factors, or pose constraints for trajectory and landmark estimation. In contrast, our method converts filtered, object-conditioned FLS returns into sparse metric depth anchors that regularize monocular depth before the resulting depth prior is used in tracking and Gaussian mapping. Compared with monocular GS-SLAM methods [14,15,31], SonarReg-GS SLAM retains the same camera-tracking and dense full-scene mapping objective while augmenting the monocular depth prior with external acoustic range cues. Compared with sonar-guided Gaussian reconstruction or novel-view synthesis methods [32,33], our method estimates camera motion and maintains a dense full-scene RGB Gaussian map rather than focusing primarily on scene reconstruction or acoustic rendering. This formulation incorporates metric sonar cues into the geometric prior while preserving the dense full-scene Gaussian mapping objective.

## 3. Preliminaries

### 3.1. 3D Gaussian Splatting

In 3D Gaussian Splatting (3DGS), a scene is represented by a set of anisotropic Gaussian primitives ($G={\left\{g_{i}\right\}}_{i=1}^{N}$). Each primitive ($g_{i}$) is parameterized by a center position ($\mu_{i}\in R^{3}$), a 3D covariance matrix ($\Sigma_{i}\in R^{3\times 3}$), an opacity value ($o_{i}\in \left[0,1\right]$), and a color vector ($c_{i}$). Following common practice in Gaussian-SLAM systems [14], we use view-independent colors and omit spherical harmonics coefficients to reduce the online optimization cost. The covariance matrix describes the anisotropic spatial extent of each Gaussian and is factorized into a scale matrix ($S_{i}\in R^{3\times 3}$) and a rotation matrix ($R_{i}\in SO\left(3\right)$):

(1) $$\Sigma_{i}=R_{i}S_{i}S_{i}^{T}R_{i}^{T}.$$

Given a world-to-camera pose ($T_{cw}=\left[R_{cw}|t_{cw}\right]$, where $R_{cw}$ and $t_{cw}$ are the rotation and translation from the world frame to the camera frame), the 3D Gaussians are projected onto the image plane through differentiable rasterization. The projected 2D covariance of Gaussian $g_{i}$ is approximated by

(2) $$\Sigma_{i}^{\prime }=J_{i}R_{cw}\Sigma_{i}R_{cw}^{T}J_{i}^{T},$$

where $J_{i}$ is the Jacobian of the local affine approximation of the perspective projection. Let $\mu_{i}^{\prime }$ be the projected 2D mean of $g_{i}$. For a pixel (*x*), the opacity contribution of $g_{i}$ is

(3) $$\alpha_{i}\left(x\right)=o_{i}\exp\left(-\frac{1}{2}{\left(x-\mu_{i}^{\prime }\right)}^{T}{\left(\Sigma_{i}^{\prime }\right)}^{-1}\left(x-\mu_{i}^{\prime }\right)\right).$$

For all Gaussians whose projections overlap with pixel *x*, sorted in front-to-back depth order, the accumulated transmittance of the *i*-th Gaussian is

(4) $$T_{i}\left(x\right)=\prod_{j=1}^{i-1}\left(1-\alpha_{j}\left(x\right)\right).$$

The rendered RGB image ($\hat{I}$) and rendered metric depth ($\hat{D}$) are obtained by alpha blending [13]:

(5) $$\hat{I}\left(x\right)=\sum_{i=1}^{N_{x}}T_{i}\left(x\right)\alpha_{i}\left(x\right)c_{i},\;\hat{D}\left(x\right)=\sum_{i=1}^{N_{x}}T_{i}\left(x\right)\alpha_{i}\left(x\right)z_{i},$$

where $N_{x}$ denotes the number of projected Gaussians contributing to pixel *x* and $z_{i}$ is the metric depth of the Gaussian center in the camera coordinate frame. These differentiable RGB and depth renderings provide the basis for the tracking and mapping objectives introduced in the following sections.

### 3.2. Forward-Looking Sonar Imaging Geometry

FLS produces polar images by emitting acoustic pulses and measuring returned echo intensity [35]. Unlike optical cameras, a 2D FLS frame (*S*) is typically recorded as a range–azimuth intensity map. Let *r* denote the acoustic range, θ denote the horizontal azimuth angle, and ϕ denote the elevation angle. Although the sonar sensing geometry has a finite elevation aperture, the recorded 2D image does not explicitly resolve the elevation angle [35]. The sensing volume can be described by

(6) $$r\in \left[r_{\min},r_{\max}\right],\;\theta \in \left[-\theta_{\max},\theta_{\max}\right],\;\phi \in \left[-\phi_{\max},\phi_{\max}\right].$$

We represent the FLS image as $S\in R^{N_{r}\times N_{\theta }}$, where $N_{r}$ and $N_{\theta }$ denote the numbers of range and azimuth bins, respectively. The range and azimuth sampling resolutions are determined by the sonar configuration, including the sensing range, field of view, and numbers of range and azimuth bins.

The key advantage of FLS is that its range coordinate is derived from acoustic time of flight and is therefore associated with metric distance. Although sonar images are visually distinct from RGB images and difficult to match directly, their range bins provide sparse metric cues that can complement monocular depth, whose scale is often unreliable in underwater scenes. We denote the Cartesian coordinate of a 3D point represented in the sonar coordinate system as $P_{s}={\left[x_{s},y_{s},z_{s}\right]}^{T}$, where $z_{s}$ is the forward range direction, $x_{s}$ is the horizontal azimuth direction, and $y_{s}$ is the vertical elevation direction. The corresponding polar representation is given by range (*r*), azimuth (θ), and elevation (ϕ). The transformation from the polar domain to the Cartesian sonar frame can be written as

(7) $$P_{s}\left(r,\theta ,\phi \right)=\left[\begin{array}{c} x_{s}\\ y_{s}\\ z_{s} \end{array}\right]=\left[\begin{array}{c} r\cos\phi \sin\theta \\ r\sin\phi \\ r\cos\phi \cos\theta \end{array}\right].$$

Equivalently, the inverse relation is

(8) $$r=\sqrt{x_{s}^{2}+y_{s}^{2}+z_{s}^{2}},\;\theta =atan2\left(x_{s},z_{s}\right),\;\phi =atan2\left(y_{s},\sqrt{x_{s}^{2}+z_{s}^{2}}\right).$$

FLS measurements are not dense depth maps: because the elevation angle is not explicitly resolved in the recorded 2D image, echoes from different elevations but with similar range and azimuth may contribute to the same range–azimuth bin. In addition, multipath, reverberation, and reflective surfaces can introduce spurious responses. Therefore, instead of inserting the raw sonar image into the Gaussian map, we extract reliable object-conditioned range anchors. These anchors provide sparse metric constraints for regularizing monocular depth in object-mask regions, while full-scene Gaussian mapping is still maintained using RGB observations and monocular depth priors. This range–azimuth geometry forms the basis for the sonar-guided depth regularization described in Section 4.2.

## 4. Method

### 4.1. Overview

Given a sequence of RGB frames and synchronized FLS images captured in underwater environments, our framework estimates the camera trajectory while reconstructing the scene as a dense 3D Gaussian map. Building on the 3DGS and FLS imaging formulations introduced in Section 3, our method constructs a sonar-regularized depth prior and integrates it into dense tracking and Gaussian mapping. Since monocular depth estimators provide dense structural cues but usually lack a reliable metric scale in underwater scenes, we use FLS measurements as sparse metric anchors to regularize monocular depth.

Specifically, Grounded-SAM object masks are used as object-level spatial priors for acoustic association. We sample near-side contour pixels from the detected object masks, associate their horizontal visual bearings with corresponding FLS azimuth beams, and extract reliable echo ranges as sparse range anchors. These anchors are used to estimate an object-conditioned scale factor for each valid object mask in a selected keyframe and construct a sonar-regularized depth prior ($D^{★}$) within the corresponding object-mask region (Section 4.2).

The corrected depth prior is incorporated into dense tracking through inverse-depth regularization in dense bundle adjustment (Section 4.3). For mapping, we follow the common Gaussian map optimization pipeline and introduce an object-aware RGB mask loss to strengthen supervision inside detected object regions without changing the dense full-scene mapping objective (Section 4.4).

### 4.2. Sonar-Guided Depth Regularization

We use FLS measurements as sparse metric constraints for regularization of monocular depth in selected keyframes. For clarity, we describe the procedure for one selected keyframe and omit the keyframe index unless necessary. Given an RGB image (*I*) and a synchronized FLS image (*S*), the module constructs a sonar-regularized depth prior ($D^{★}$).

**Object-mask prior and near-side contour sampling.** Given an RGB image (*I*), Grounded-SAM extracts target object masks according to the target prompt. If multiple target masks are detected, we process each mask independently to avoid mixing acoustic anchors from objects at different ranges. For clarity, we describe the following procedure for one object mask and denote it as $M^{obj}$. The mask serves as an object-level spatial prior for acoustic association rather than as a dense semantic map.

We obtain an initial dense monocular depth map ($\tilde{D}$) using the Metric3D V2 depth estimator [18]. Although $\tilde{D}$ provides dense structural cues, its metric scale can be unreliable in underwater scenes. Therefore, we use $\tilde{D}$ mainly to select candidate acoustic queries and use FLS measurements to provide metric-scale constraints.

Specifically, we first extract a boundary band (*B*) from $M^{obj}$ and compute a near-side depth threshold ($d_{\tau }$) from the depth values on the boundary. The near-side contour samples are selected as

(9) $$C=\left\{m\in B|\tilde{D}\left(m\right)\leq d_{\tau }\right\},$$

where $d_{\tau }$ is the τ-th percentile of $\left\{\tilde{D}\left(q\right)|q\in B\right\}$. This contour-biased sampling is motivated by the fact that FLS echoes are often dominated by the closest visible or strongly reflective surface of the object.

**Opti-acoustic association and range-anchor extraction.** We compute the horizontal visual bearing for each sampled mask-contour coordinate ($m=(u_{m},v_{m})\in C$) from the camera intrinsics:

(10) $$\theta \left(m\right)=\arctan\left(\frac{u_{m}-c_{x}}{f_{x}}\right),$$

where $f_{x}$ and $c_{x}$ denote the horizontal focal length and principal point of the RGB camera intrinsic matrix, respectively.

Since the camera and FLS are mounted in a co-located and approximately aligned configuration, we use θ(m) to query the corresponding azimuth beam in the FLS image. For more general camera–sonar rigs, this association would require explicit extrinsic calibration. Along this associated beam, we apply a Constant False-Alarm Rate (CFAR)-based detector to extract a statistically significant echo response and use its range as the acoustic anchor. The CFAR estimates an adaptive threshold from neighboring reference cells, making it suitable for sonar images with spatially varying reverberation [36,37].

Under the co-located and approximately aligned sensor setup, we use the extracted FLS range as an approximate metric anchor to correct the scale of the monocular depth in the corresponding object region.

Candidate anchors are further filtered by local consistency in the bearing-range space, since valid object echoes usually form a locally smooth contour in the FLS image. The retained valid range-anchor set is denoted as

(11) $$A=\left\{\left(m_{i},r_{i}^{s}\right)|m_{i}\in C,\;r_{i}^{s}\;\mathrm{is}\;\mathrm{valid}\right\}.$$

Here, $r_{i}^{s}$ denotes the FLS range extracted from the azimuth beam associated with $\theta (m_{i})$.

**Scale estimation and regularized depth prior.** Under the approximately co-located and aligned camera–FLS setup, we use the monocular depth values at the associated contour samples as approximate range proxies for scale estimation.

For this object mask, we estimate a scale factor by comparing the retained FLS ranges with the monocular depth values at the corresponding sampled coordinates:

(12) $$\gamma =\frac{{\mathrm{median}}_{i}\left(r_{i}^{s}\right)}{{\mathrm{median}}_{i}\left(\tilde{D}\left(m_{i}\right)\right)+\epsilon },\;\left(m_{i},r_{i}^{s}\right)\in A.$$

Here, ${\mathrm{median}}_{i}\left(\cdot \right)$ is computed over all valid anchor pairs ($(m_{i},r_{i}^{s})\in A$), and ϵ is a small constant for numerical stability.

The corrected depth prior is obtained by applying this scale inside the detected object-mask region:

(13) $$D^{★}\left(p\right)=\left\{\begin{array}{cc} \gamma \tilde{D}\left(p\right), & p\in M^{obj},\\ \tilde{D}\left(p\right), & p\notin M^{obj}. \end{array}\right.$$

When multiple target masks are present, the same procedure is applied independently to each mask to avoid mixing anchors from objects at different ranges. For keyframes without sufficient reliable anchors, the system falls back to the original monocular depth prior. The resulting prior for keyframe *k* is denoted as $D_{k}^{★}$ when used in the tracking and mapping modules.

It should be noted that the proposed sonar regularization does not directly impose a global trajectory-scale constraint. Instead, it provides local metric depth anchors in object-mask regions, which are mainly used to improve Gaussian initialization and depth supervision for mapping and are also incorporated as regularized depth priors during dense tracking.

### 4.3. Dense Tracking with Sonar-Regularized Depth Prior

Underwater image sequences often contain weak texture, reduced contrast, and medium-induced appearance changes, which make sparse feature matching unstable. We therefore adopt a dense tracking architecture inspired by DROID-SLAM [38]. The tracker estimates dense pixel-wise correspondences between covisible frames and refines them recurrently, providing stronger constraints for pose estimation than sparse keypoints.

**Figure 1:**
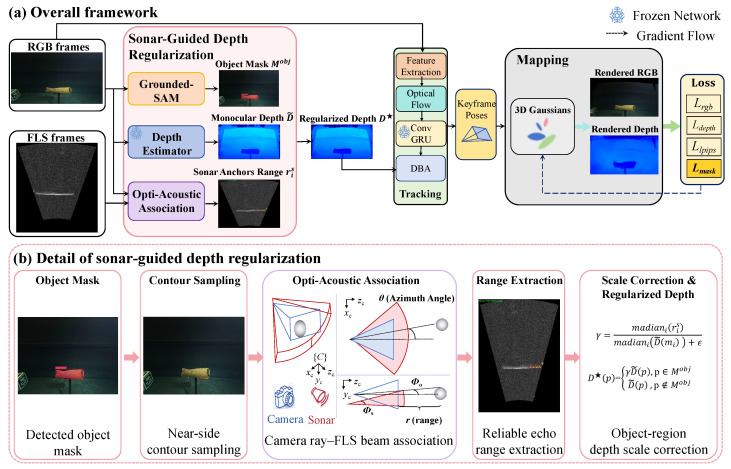
**Overview of the proposed SonarReg-GS SLAM framework.** The system takes synchronized RGB and FLS frames as input. RGB images provide object masks and monocular depth priors, while FLS images provide sparse metric range cues. Object-conditioned sonar anchors are extracted through near-side contour sampling, camera–sonar beam association, and echo range filtering. The extracted anchors regularize monocular depth in object-mask regions and provide metric depth priors for tracking and dense Gaussian mapping. An object-aware RGB mask loss further increases supervision in detected object regions while preserving full-scene Gaussian mapping.

As shown in Figure 1, the tracking module follows a feature extraction–optical flow–recurrent refinement–dense optimization pipeline. Specifically, a Convolutional Gated Recurrent Unit (ConvGRU) is used for correspondence refinement, and a Dense Bundle Adjustment (DBA) layer is used for joint pose and inverse-depth optimization. The tracking problem is formulated on an active covisibility graph (G=(V,E), where *V* denotes the selected keyframes and E denotes the pairwise frame connections used for optimization). For each connected keyframe pair, dense image features are first extracted and used by the optical-flow update module to estimate pixel-wise correspondences across frames. The ConvGRU [38] recurrently refines these correspondences and predicts their confidence weights. The refined correspondences are then passed to the DBA layer, which jointly optimizes keyframe poses and inverse-depth maps.

Let $\omega_{i}$ denote the camera-to-world pose of keyframe *i*, and let $d_{i}$ denote the optimized inverse-depth map. We incorporate the sonar-regularized depth prior into DBA as an inverse-depth regularization term:

(14) $$\begin{array}{ll} E(\omega ,d)= & \sum_{(i,j)\in E}\sum_{p\in \Omega_{i}}{∥p_{ij}^{*}\left(p\right)-\Pi_{c}\left(\omega_{j}^{-1}\omega_{i}\Pi_{c}^{-1}\left(p,d_{i}\left(p\right)\right)\right)∥}_{\Sigma_{ij}\left(p\right)}^{2}\\ & +\lambda_{d}\sum_{i\in V}\sum_{p\in \Omega_{i}}W_{i}\left(p\right){∥d_{i}\left(p\right)-{\left(D_{i}^{★}\left(p\right)\right)}^{-1}∥}_{2}^{2}. \end{array}$$

Here, $\Omega_{i}$ denotes the set of pixels used for dense tracking, and $\Pi_{c}$ and $\Pi_{c}^{-1}$ are camera projection and back-projection operators, respectively.

The first term minimizes the dense reprojection residual induced by the recurrently refined correspondences. ${∥\cdot ∥}_{\Sigma_{ij}\left(p\right)}^{2}$ denotes the Mahalanobis distance, where $\Sigma_{ij}\left(p\right)$ represents the confidence weights predicted by the optical-flow update module. The second term regularizes the optimized inverse depth using the sonar-regularized keyframe depth prior ($D_{i}^{★}$). Here, $\lambda_{d}$ denotes the depth-supervision coefficient of the DROID-SLAM-based dense bundle adjustment backend, and $W_{i}\left(p\right)$ is a validity weight that disables the regularization where the prior is invalid or unreliable.

In contrast with directly using the initial monocular depth (${\tilde{D}}_{i}$), the prior ($D_{i}^{★}$) contains object-region scale correction from reliable FLS range anchors. In this way, acoustic measurements influence tracking indirectly through the regularized depth prior rather than being introduced as explicit sonar reprojection or point-matching residuals in the pose optimization. This design preserves the dense visual tracking formulation while introducing sparse metric-scale information from FLS measurements.

### 4.4. Gaussian Mapping with Object-Aware Mask Supervision

After the tracking module estimates the pose of a newly inserted keyframe, its RGB image (*I*), sonar-regularized depth prior ($D^{★}$), and camera pose are forwarded to the mapping module. The mapping module maintains a full-scene 3D Gaussian map and updates it incrementally as new keyframes are processed.

Following common Gaussian-SLAM practice, the depth prior is used as proxy geometry to initialize or expand Gaussian primitives in newly observed regions, while the map parameters are refined by differentiable RGB and depth rendering.

For each selected keyframe, we render an RGB image ($\hat{I}$) and a metric depth map ($\hat{D}$) from the current Gaussian map at the corresponding camera pose. The observed RGB image (*I*) provides full-image photometric supervision, while the sonar-regularized depth prior ($D^{★}$) constrains the rendered depth. Therefore, the mapping objective is first defined over the full image to preserve dense full-scene reconstruction.

The RGB reconstruction loss combines photometric and structural similarity terms:

(15) $$L_{rgb}=\left(1-\lambda_{ssim}\right){∥\hat{I}-I∥}_{1}+\lambda_{ssim}\left(1-\mathrm{SSIM}(\hat{I},I)\right),$$

which provides full-image appearance supervision for Gaussian color, opacity, and geometric optimization. The depth consistency loss constrains the rendered depth using the sonar-regularized depth prior:

(16) $$L_{depth}={∥\hat{D}-D^{★}∥}_{1}.$$

Underwater target objects often occupy limited image regions and can be weakly constrained by global photometric losses, especially under low contrast, turbidity, and background clutter. To strengthen the reconstruction of detected target objects without changing the full-scene mapping objective, we introduce an object-aware RGB mask loss that provides additional photometric supervision inside the object-mask regions, defined as

(17) $$L_{mask}=\frac{{∥M^{obj}⊙\left(\hat{I}-I\right)∥}_{1}}{\sum_{p}M^{obj}\left(p\right)+\epsilon },$$

where ⊙ denotes element-wise multiplication and ϵ is a small constant for numerical stability.

This term is not a semantic segmentation loss and does not restrict mapping to the object region. Instead, it adds region-level photometric supervision inside the detected object mask, improving the reconstruction of target objects while preserving the dense full-scene Gaussian mapping objective.

We also use a perceptual loss to improve perceptual consistency:

(18) $$L_{lpips}=\mathrm{LPIPS}\left(\hat{I},I\right).$$

In addition, an isotropic scale regularization term ($L_{iso}$) is applied to penalize large-scale differences among the three axes of each Gaussian primitive, preventing excessively elongated or flattened Gaussians during map optimization. The final mapping objective is written as follows:

(19) $$L_{map}=\lambda_{rgb}L_{rgb}+\lambda_{depth}L_{depth}+\lambda_{mask}L_{mask}+\lambda_{lpips}L_{lpips}+\lambda_{iso}L_{iso},$$

where the scalar weights balance the contribution of each term. The RGB, perceptual, and isotropic regularization terms follow common Gaussian mapping practice, while $L_{depth}$ and $L_{mask}$ incorporate the proposed sonar-regularized depth prior and object-aware supervision.

## 5. Experiments

### 5.1. Experimental Setup

**Datasets.** We evaluate the proposed SonarReg-GS SLAM on two public underwater RGB–sonar datasets collected in real-world sensing setups: the UXO dataset [12] and the OPTI dataset [11]. Both datasets provide synchronized optical and acoustic observations, making them suitable for evaluating whether sparse sonar measurements can serve as metric constraints for underwater monocular GS-SLAM.

The UXO dataset [12] is an acoustic–optical dataset collected for underwater unexploded ordnance perception. The data were captured in a controlled test basin using an ARIS Explorer 3000 imaging sonar (Sound Metrics Corporation, Bellevue, WA, USA) and a HERO8 Black camera (GoPro, Inc., San Mateo, CA, USA) mounted on a pan-tilt unit attached to a gantry crane. The dataset contains multiple UXO-like targets, including a 100 lb general purpose bomb, a phosphor bomb, a mortar bomb, and a test cylinder. It provides raw and polar-transformed sonar frames, matched camera frames, calibration parameters, target information, and sensor metadata. These sequences contain metallic objects, strong acoustic reflections, low-texture regions, and underwater appearance variations, which make them challenging for purely visual SLAM and suitable for evaluating sonar-guided metric regularization. In the experimental results, we use the original recording-oriented sequence names to identify the evaluated UXO sequences. Specifically, “100lbs_bomb” denotes a selected recording of the 100 lb general-purpose bomb. “15cm_mortar” denotes a selected mortar sequence with a propellant charge case and tail fins. “20 lbs” denotes a selected recording of a deformed phosphor bomb. “100lbs_bomb_floor” denotes a recording of the 100 lb bomb placed on the basin floor, and “test_cylinder” denotes a selected recording of the reference cylinder. These names are used consistently in the quantitative tables and qualitative figures.

The OPTI dataset [11] is an underwater opti-acoustic semantic SLAM dataset collected with a monocular camera, multibeam sonar, an IMU, a DVL, and a pressure sensor. It contains indoor and outdoor underwater sequences with challenging acoustic and lighting conditions. The dataset provides reference trajectories obtained from a low-cost Hybrid Long/Inverted Ultra-Short Baseline (iLBL-USBL) acoustic beacon system, enabling quantitative evaluation of trajectory accuracy. We use three OPTI sequences, denoted as Run1, Run2, and Run3, to evaluate the robustness of different SLAM systems under real underwater opti-acoustic sensing conditions.

**Reference Trajectories.** For the OPTI dataset, we directly use the provided reference trajectories for localization evaluation. For the UXO dataset, ready-to-use per-frame camera-trajectory ground truth is not provided in the format required by our SLAM evaluation pipeline. Therefore, we reconstruct reference camera trajectories using COLMAP 3.10-dev [39]. Following common practice in underwater SLAM evaluation [7,40], these SfM-reconstructed trajectories are used as pseudo reference trajectories for quantitative comparison.

**Baselines.** We compare SonarReg-GS SLAM with representative classical and Gaussian-based SLAM methods, including ORB-SLAM3 [27], MonoGS [14], and Splat-SLAM [31]. ORB-SLAM3 is included as a classical feature-based SLAM baseline. MonoGS and Splat-SLAM are selected as representative monocular Gaussian splatting SLAM systems. For ORB-SLAM3 and MonoGS, we evaluate both depth-free and depth-enabled variants when applicable. The depth-free setting uses only monocular RGB input, while the depth-enabled setting incorporates estimated monocular depth priors. For depth-enabled variants, we use the same monocular depth estimator [18] as the initial depth source, without sonar-based scale regularization. Splat-SLAM is evaluated under its default depth-prior setting, since it relies on monocular depth priors in its standard pipeline. Our method further uses sonar-derived metric cues to regularize the depth scale during tracking and Gaussian mapping.

Existing underwater visual–acoustic SLAM systems, such as Opti-Acoustic Semantic SLAM [11] and SVIn2 [4], are considered as related work rather than direct quantitative baselines because their sensing assumptions and output representations are different from ours. These systems rely on additional sensors such as IMU, DVL, and pressure/depth sensors, which provide motion, scale, or depth constraints beyond the RGB-FLS setting considered in this work. Moreover, they mainly produce trajectory or landmark estimates, whereas ours targets dense full-scene Gaussian mapping with rendering-based evaluation.

**Metrics.** We report the Absolute Trajectory Error (ATE) RMSE to evaluate tracking accuracy. Before computing the error, each estimated trajectory is aligned with the reference trajectory using Sim(3) Umeyama alignment. This metric evaluates trajectory consistency after global similarity alignment. Since our sonar-guided depth regularization is applied locally within object-mask regions, the Sim(3)-aligned ATE is used to assess tracking robustness rather than to independently verify global scale accuracy. The metric-scale effect of sonar anchors is instead reflected through the object-region depth regularization process and the rendering-based mapping results. For mapping quality, we evaluate the rendered images using PSNR, SSIM [41], and LPIPS [42]. PSNR reflects pixel-wise reconstruction fidelity; a higher value indicates better rendering quality. SSIM measures structural consistency between the rendered and reference images, with larger values corresponding to better structural similarity. LPIPS evaluates perceptual discrepancy in a learned feature space; a lower score indicates higher perceptual similarity.

**Implementation Details.** All experiments are conducted on a workstation with an Intel Core i9-14900KF CPU, 32 GB RAM, and an NVIDIA RTX 4090 GPU. The system runs Ubuntu 22.04 LTS, and the implementation uses Python 3.10.18, PyTorch 2.1.0, and CUDA 11.8. All compared methods are evaluated under the same hardware environment.

### 5.2. Visualization of Sonar-Guided Depth Regularization

Before presenting quantitative tracking and mapping results, we first visualize the proposed sonar-guided depth regularization process. Since dense ground-truth depth is unavailable in the evaluated datasets, this visualization is intended to illustrate the RGB–sonar association and depth-scale correction process rather than to evaluate depth accuracy.

**Figure 2:**
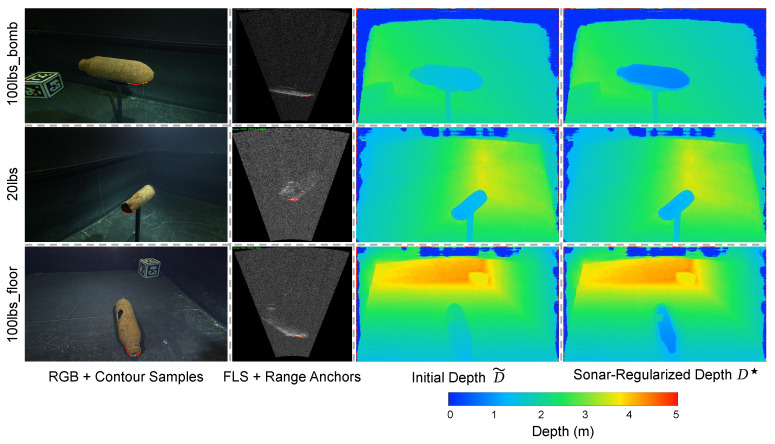
**Visualization of sonar-guided depth regularization.** We show three representative UXO sequences from top to bottom. For each sequence, the columns show the RGB image with sampled near-side object contours, the corresponding FLS image with selected acoustic range anchors, the initial monocular depth ($\tilde{D}$), and the sonar-regularized depth prior ($D^{★}$). The selected FLS anchors provide sparse metric-range cues for estimating an object-region depth-scale factor. Compared with the initial monocular depth, the regularized depth prior shows structured scale adjustment in the detected object region. This visualization illustrates the regularization process qualitatively; it is not intended as a quantitative depth-accuracy evaluation because dense ground-truth depth is unavailable. Therefore, the visualization does not claim that $D^{★}$ is directly more accurate than $\tilde{D}$. The utility of the regularized depth prior is instead evaluated indirectly through its downstream effects on tracking and Gaussian mapping.

Figure 2 shows representative examples from the UXO dataset. The RGB images provide object masks and near-side contour samples, which define the visual support for acoustic association. For these sampled pixels, the corresponding FLS azimuth beams are queried to extract valid range anchors after echo filtering and local consistency checking. The extracted anchors are then used to estimate an object-region scale factor and construct the sonar-regularized depth prior.

Compared with the initial monocular depth ($\tilde{D}$), the sonar-regularized depth prior ($D^{★}$) shows structured scale adjustment in the detected object regions. This indicates that sonar measurements are not directly inserted as dense geometry; instead, they are used as sparse metric constraints to regularize monocular depth before tracking and Gaussian mapping.

Because dense ground-truth depth is unavailable, this visualization does not establish that $D^{★}$ is directly more accurate than $\tilde{D}$. Instead, $D^{★}$ is treated as a sonar-constrained depth prior for the downstream SLAM pipeline. Its practical effectiveness in improving tracking and Gaussian mapping is evaluated quantitatively in the subsequent experiments and further verified through the ablation study.

### 5.3. Tracking Results

**Figure 3:**
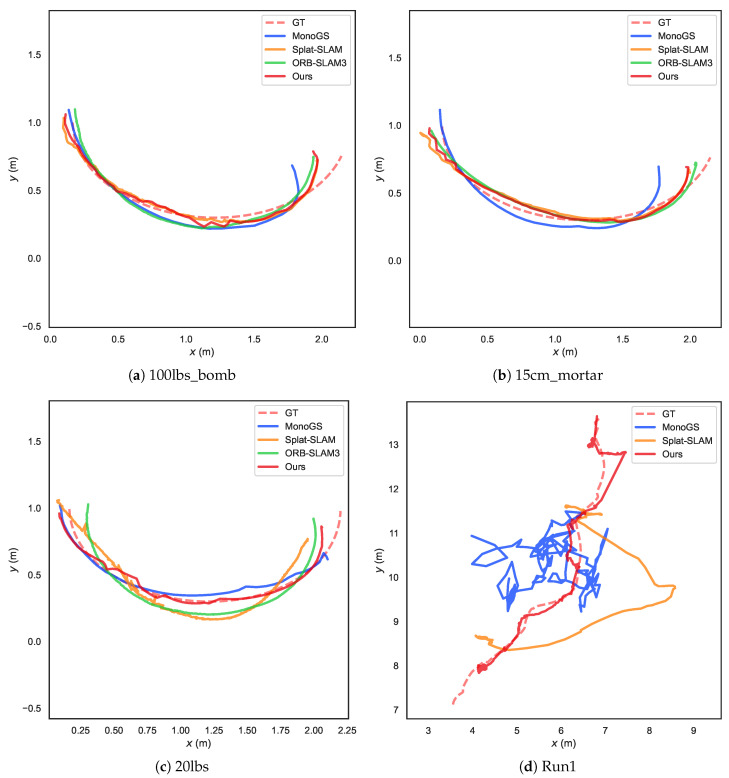
**Representative trajectory visualizations on the underwater RGB–FLS datasets.** Subfigures (**a**–**c**) show UXO sequences of a 100 lb bomb, 15 cm mortar, and 20 lb bomb, respectively, while subfigure (**d**) shows the OPTI Run1 sequence. ORB-SLAM3 fails to track Run1 and is therefore not plotted in subfigure (**d**).

Table 1 reports the tracking accuracy on the UXO dataset; representative trajectories are visualized in Figure 3a–c. Our method achieves the lowest average ATE RMSE of 0.1015 m, outperforming ORB-SLAM3, MonoGS, and Splat-SLAM. Compared with the Gaussian-based SLAM baseline, Splat-SLAM, our method reduces the average ATE by 21.7%. Moreover, our method ranks first or second on all five sequences, showing more consistent tracking performance across different UXO objects and scene layouts.

It is worth noting that directly introducing monocular depth does not consistently improve tracking. For example, MonoGS with depth obtains a higher average ATE than its depth-free variant. This suggests that monocular depth priors in underwater scenes can still suffer from scale errors and scene-dependent bias. In contrast, our sonar-guided regularization provides an additional depth prior for dense tracking using sparse but metrically grounded FLS range anchors.

Table 2 reports the tracking accuracy on the OPTI dataset; a representative trajectory is visualized in Figure 3d. ORB-SLAM3 fails on all sequences under both depth-free and depth-enabled settings, indicating that OPTI poses significant challenges to traditional feature-based visual SLAM. Among the successfully tracked methods, our method achieves the lowest average ATE RMSE of 0.4640 m, reducing the average error by 18.4% compared with Splat-SLAM and by 49.1% compared with MonoGS without depth.

Similarly, directly incorporating monocular depth does not improve MonoGS on this dataset. MonoGS with depth obtains a higher average ATE than its depth-free variant, suggesting that monocular depth priors can introduce scale bias and scene-dependent errors in underwater environments. Our method does not obtain the best result on every individual OPTI sequence, but it achieves the lowest average ATE, suggesting that sonar-guided depth regularization provides useful constraints for overall tracking performance.

### 5.4. Mapping Results

**Figure 4:**
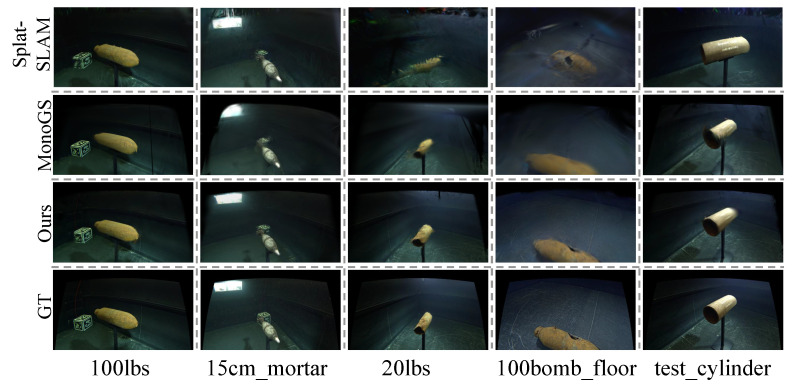
**Rendering visualization on the UXO dataset.** Compared with monocular Gaussian-SLAM baselines, our method shows cleaner object appearance and fewer visible rendering artifacts by using sonar-regularized depth priors for Gaussian mapping. The black peripheral regions originate from the distortion correction of the underwater camera images and are excluded from the qualitative interpretation.

Table 3 reports the quantitative rendering results on the UXO dataset, while Figure 4 provides the corresponding qualitative comparisons. Our method achieves the best average PSNR of 29.61 dB and the lowest average LPIPS of 0.173. Specifically, our method obtains the best PSNR and LPIPS on all five sequences, indicating improved photometric fidelity and perceptual rendering quality. Compared with MonoGS with depth, our method improves the average PSNR by 1.56 dB and reduces LPIPS by 52.3%.

Although our method does not achieve the highest average SSIM, it maintains competitive structural similarity while substantially improving PSNR and LPIPS. This result suggests that the sonar-regularized depth prior provides useful supervision for Gaussian mapping, especially in terms of photometric fidelity and perceptual quality.

**Figure 5:**
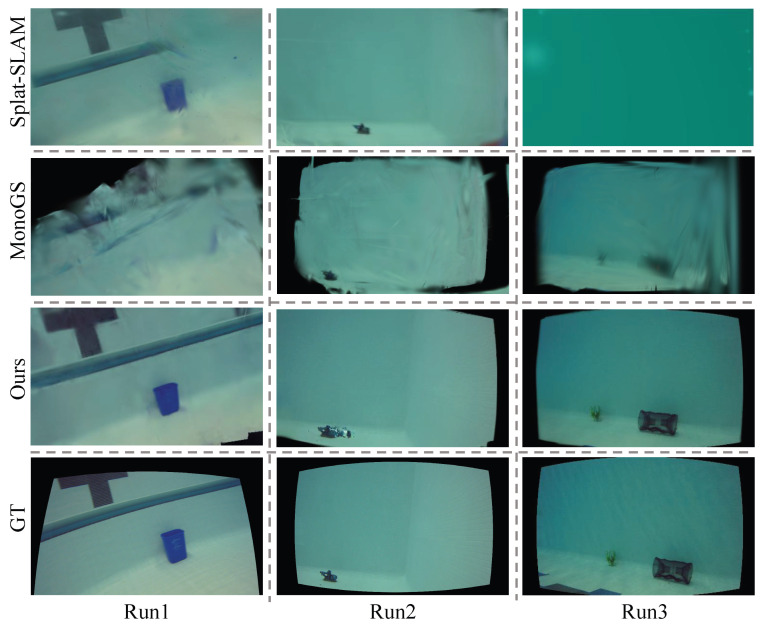
**Rendering visualization on the OPTI dataset.** Our method shows improved rendering fidelity under challenging underwater appearance changes by combining RGB reconstruction with sonar-regularized depth supervision. The black peripheral regions originate from the distortion correction of the underwater camera images and are excluded from the qualitative interpretation.

Table 4 reports the quantitative rendering results on the OPTI dataset, while Figure 5 provides the corresponding qualitative comparisons. Our method achieves the best average PSNR of 28.63 dB and the lowest average LPIPS of 0.303 while obtaining a comparable average SSIM. In particular, our method improves the average PSNR from 20.91 dB to 28.63 dB compared with MonoGS without depth and reduces the average LPIPS from 0.450 to 0.303. Compared with Splat-SLAM, our method improves the average PSNR by 10.03 dB and reduces LPIPS by 52.9%.

These rendering results suggest that sonar-regularized depth supervision improves Gaussian mapping quality, especially in terms of PSNR and LPIPS. Although MonoGS without depth achieves a slightly higher SSIM on some individual runs, our method consistently obtains the best PSNR and LPIPS on all three sequences, suggesting better photometric fidelity and perceptual rendering quality.

### 5.5. Robustness Analysis

**Robustness to Object-Mask Perturbations.** We evaluate the robustness of the proposed association strategy to two common types of segmentation error: mask-boundary variation and spatial localization error. The experiments are conducted on the 100lbs_bomb sequence. For mask-boundary variations, the detected masks are eroded or dilated by 5 and 10 pixels. For localization errors, the entire masks are shifted horizontally or vertically by ±5 and ±10 pixels. For each spatial-shift setting, the results from the two opposite directions along the same axis are averaged. Only the mask input is perturbed, while all other system components and parameters remain unchanged.

As shown in Table 5, the proposed method remains stable under the tested perturbations. Across all perturbed settings, the ATE RMSE ranges from 0.0614 m to 0.0700 m, the PSNR from 30.25 dB to 30.84 dB, the SSIM from 0.881 to 0.891, and the LPIPS from 0.115 to 0.126. These results are comparable to those obtained with the original mask, which achieves an ATE RMSE of 0.0624 m, a PSNR of 30.95 dB, an SSIM of 0.886, and an LPIPS of 0.121.

Among the tested localization perturbations, horizontal shifts produce the largest increase in ATE RMSE. This observation is consistent with the association mechanism because horizontal displacement directly changes the visual bearing used to query the corresponding FLS beam. Nevertheless, even under a horizontal shift of ±10 pixels, the method achieves an ATE RMSE of 0.0700 m and a PSNR of 30.40 dB without tracking failure. The observed stability is consistent with the use of near-side contour sampling, bearing-to-beam gating, echo-response filtering, local consistency checking, valid-pair verification, and median-based scale estimation, which reduce the influence of moderate mask inaccuracies.

Overall, the results indicate that the proposed association strategy tolerates moderate mask-boundary and localization errors without substantial degradation in tracking or rendering performance.

**Robustness to Camera–Sonar Alignment Errors.** Robustness evaluation is important for the practical deployment of robotic perception and SLAM systems [43,44].

In our setup, the camera and FLS are mounted in a co-located and approximately aligned configuration, allowing the horizontal visual bearing to be directly used for querying of the corresponding FLS azimuth beam. We therefore evaluate the sensitivity of SonarReg-GS SLAM to residual yaw misalignment between the two sensors.

Specifically, synthetic yaw offsets of $\pm 2^{\circ }$, $\pm 5^{\circ }$, and $\pm 10^{\circ }$ are introduced into the bearing-to-beam association, while all other components and parameters remain unchanged. Results for positive and negative offsets with the same magnitude are averaged.

As shown in Table 6, the proposed method shows limited performance variation under the tested perturbations. The ATE RMSE remains within the range of 0.0631–0.0681 m, and PSNR values fall within the range of 30.46–30.66 dB. The SSIM remains at 0.883, while the LPIPS ranges from 0.120 to 0.122. Even at an absolute yaw offset of $10^{\circ }$, the method achieves an ATE RMSE of 0.0665 m and a PSNR of 30.66 dB.

These results indicate reasonable robustness to residual yaw misalignment under the approximately aligned sensor configuration.

**Parameter Sensitivity.** We conduct one-factor-at-a-time experiments on the 100lbs_bomb sequence for the mask-loss weight, sonar intensity threshold, and RGB–FLS association beam window.

The sonar intensity threshold filters weak echo responses: a candidate range anchor is retained only when the strongest sampled echo response is no lower than the threshold, whose default value is 0.15. For the association sensitivity analysis, the beam window specifies the number of adjacent FLS beams searched on each side of the beam predicted from the visual bearing. The maximum response within this window is used for range extraction, and the default value of 0 denotes nearest-beam association.

Each parameter is varied independently while all other settings remain unchanged. The main setting serves as the common reference point for the three parameter sweeps.

As shown in Table 7, the method remains stable over the tested parameter ranges. Varying the mask-loss weight results in an ATE RMSE of 0.0623–0.0697 m and a PSNR of 30.34–30.95 dB. Changing the sonar intensity threshold yields an ATE RMSE of 0.0624–0.0714 m and a PSNR of 30.40–30.95 dB. Similarly, varying the association beam window from 0 to 5 produces an ATE RMSE of 0.0624–0.0648 m and a PSNR of 30.53–30.95 dB. Overall, the tracking and rendering metrics show limited variation over the tested parameter ranges. This indicates that the proposed method has limited sensitivity to moderate variations in the evaluated parameters. The default settings used in the main experiments provide a practical balance between object-region supervision, acoustic-anchor reliability, and anchor availability rather than optimizing a single evaluation metric.

### 5.6. Ablation Study

We conduct an ablation study on the 100lbs_bomb sequence to evaluate the effects of the sonar-guided depth-scale correction and the object-aware mask loss. As shown in Table 8, removing the depth-scale correction increases the ATE RMSE from 0.0624 m to 0.0642 m and decreases the PSNR from 30.95 dB to 29.45 dB. This result suggests that FLS range anchors provide useful scale cues for monocular depth regularization, especially in terms of improving rendering quality in this sequence.

Removing the object-aware mask loss also degrades rendering quality, reducing PSNR from 30.95 dB to 30.28 dB and slightly worsening SSIM and LPIPS. This suggests that the proposed mask loss strengthens photometric supervision inside detected object regions and helps preserve target appearance without changing the full-scene mapping objective. Overall, the full model achieves the best results on this ablation sequence, supporting the complementary effects of sonar-guided depth regularization and object-aware mask supervision.

## 6. Limitations and Future Work

SonarReg-GS SLAM uses FLS measurements as sparse metric constraints for depth regularization rather than directly treating raw sonar observations as dense geometry. The current framework is evaluated on public RGB-sonar datasets that contain object-centric scenes and synchronized optical-acoustic observations. The sonar-anchor extraction assumes that detected object masks provide reliable visual supports and that the corresponding acoustic responses are distinguishable in the FLS range–azimuth image. When multiple objects lie along similar azimuth beams or when objects are vertically separated in the RGB image but collapsed into similar FLS range–azimuth bins, the elevation ambiguity of FLS may lead to mixed or incorrect range anchors. In addition, sonar-guided regularization requires at least one reliably detected object region with valid acoustic returns; otherwise, the system falls back to the original monocular depth prior or the latest valid scale estimate.

Several extensions could further improve the robustness and applicability of SonarReg-GS SLAM. First, since sonar-anchor extraction depends on object masks, future work will use temporal mask propagation and mask uncertainty filtering to improve object support stability across frames. Second, because FLS measurements may contain weak echoes, multipath responses, and reverberation, future work will introduce uncertainty-aware acoustic association to weight sonar anchors according to echo confidence and local geometric consistency. Third, the current RGB–FLS association assumes an approximately aligned sensor setup; a more general version will explicitly model camera–sonar extrinsics and sonar beam geometry to better handle different sensor configurations and vertically ambiguous object layouts. Finally, since foundation-model-based mask extraction and depth estimation introduce computational overhead, future work will explore more efficient online implementations for practical underwater deployment.

## 7. Conclusions

This paper introduced SonarReg-GS SLAM, a sonar-guided depth regularization framework for underwater Gaussian splatting SLAM with RGB–sonar inputs. Instead of directly inserting raw sonar observations into the Gaussian map, the proposed method extracts object-conditioned sparse sonar anchors and uses them to regularize monocular depth scale. The resulting sonar-regularized depth priors are incorporated into dense tracking and Gaussian mapping, while an object-aware RGB mask loss provides additional supervision for detected object regions without changing the full-scene mapping objective. Experiments on two public underwater RGB–sonar datasets show that SonarReg-GS SLAM improves tracking accuracy and rendering-based mapping quality compared with representative classical and Gaussian splatting SLAM baselines. Ablation results on a representative sequence further support the roles of sonar-guided depth-scale regularization and object-aware mask supervision. These results indicate that sparse FLS measurements can provide useful metric constraints for underwater Gaussian splatting SLAM when they are filtered and incorporated through depth regularization. Future work will focus on extending the framework to more diverse underwater environments, improving the robustness of object-conditioned acoustic association, and exploring more efficient online implementations.

## Figures and Tables

**Table 1:** **Tracking performance on the UXO dataset** (ATE RMSE ↓ [m]); ↓ indicates that lower values are better. The best results are shown in bold and denoted by first, while the second-best results are denoted by second.

Method	Depth	100lbs_bomb	15cm_mortar	20 lbs	100lbs_bomb_floor	test_cylinder	Avg.
ORB-SLAM3 [27]	w/o	0.5550	**0.0480**	0.1996	0.6847	**0.0020**	0.2979
	w/	0.0817	0.1206	0.1073	0.3663	0.0854	0.1523
MonoGS [14]	w/o	0.1250	0.1120	0.0917	0.4522	0.0736	0.1709
	w/	0.1250	0.1505	0.1641	0.4426	0.1010	0.1966
Splat-SLAM [31]	w/	**0.0585**	0.0765	0.1598	0.3100	0.0432	0.1296
Ours	w/	0.0624	0.0547	**0.0668**	**0.2856**	0.0378	**0.1015**

**Table 2:** **Tracking performance on the OPTI dataset** (ATE RMSE ↓ [m]); ↓ indicates that lower values are better. The best results are shown in bold and denoted by first, while the second-best results are denoted by second. ‘F’ denotes tracking failure.

Method	Depth	OPTI Dataset			
		**Run1**	**Run2**	**Run3**	**Avg.**
ORB-SLAM3 [27]	w/o	F	F	F	-
	w/	F	F	F	-
MonoGS [14]	w/o	1.6500	0.3650	0.7210	0.9120
	w/	2.0410	0.7044	0.9680	1.2378
Splat-SLAM [31]	w/	1.2790	**0.1690**	**0.2580**	0.5687
Ours	w/	**0.4510**	0.3130	0.6280	**0.4640**

**Table 3:** **Rendering performance on the UXO dataset**. ↑: higher is better; ↓: lower is better. The best results are shown in bold and denoted by first, while the second-best results are denoted by second.

Method	Depth	Metric	UXO Dataset					
			**100lbs_bomb**	**15cm_mortar**	**20 lbs**	**100lbs_bomb_floor**	**test_cylinder**	**Avg.**
MonoGS [14]	w/o	PSNR ↑	29.74	27.65	28.60	26.40	27.39	27.96
		SSIM ↑	0.877	0.834	**0.892**	**0.800**	0.839	0.848
		LPIPS ↓	0.247	0.372	0.275	0.528	0.305	0.345
	w/	PSNR ↑	29.20	27.68	27.73	27.26	28.38	28.05
		SSIM ↑	**0.886**	0.828	0.850	**0.800**	**0.883**	**0.849**
		LPIPS ↓	0.260	0.392	0.345	0.502	0.317	0.363
Splat-SLAM [31]	w/	PSNR ↑	27.70	27.02	24.26	26.20	27.79	26.59
		SSIM ↑	0.699	0.669	0.624	0.622	0.703	0.663
		LPIPS ↓	0.294	0.391	0.414	0.545	0.339	0.397
Ours	w/	PSNR ↑	**30.95**	**30.21**	**29.34**	**28.44**	**29.12**	**29.61**
		SSIM ↑	**0.886**	**0.854**	0.855	0.731	0.852	0.836
		LPIPS ↓	**0.121**	**0.169**	**0.166**	**0.236**	**0.171**	**0.173**

**Table 4:** **Rendering performance on the OPTI dataset**. ↑: higher is better; ↓: lower is better. The best results are shown in bold and denoted by first, while the second-best results are denoted by second.

Method	Depth	Metric	OPTI Dataset			
			**Run1**	**Run2**	**Run3**	**Avg.**
MonoGS [14]	w/o	PSNR ↑	19.49	20.66	22.58	20.91
		SSIM ↑	0.673	**0.837**	**0.837**	0.782
		LPIPS ↓	0.546	0.400	0.403	0.450
	w/	PSNR ↑	17.45	20.09	20.73	19.42
		SSIM ↑	0.724	0.763	0.740	0.742
		LPIPS ↓	0.510	0.470	0.478	0.486
Splat-SLAM [31]	w/	PSNR ↑	17.03	22.49	16.28	18.60
		SSIM ↑	0.537	0.717	0.597	0.617
		LPIPS ↓	0.717	0.594	0.622	0.644
Ours	w/	PSNR ↑	**28.52**	**28.78**	**28.58**	**28.63**
		SSIM ↑	**0.734**	0.815	0.796	**0.782**
		LPIPS ↓	**0.421**	**0.229**	**0.259**	**0.303**

**Table 5:** **Robustness to object-mask perturbations.** Erosion and dilation simulate mask-boundary errors, while horizontal and vertical shifts simulate localization errors. Results for opposite shift directions along the same axis are averaged. ↑: higher is better; ↓: lower is better.

Perturbation Type	Setting	ATE RMSE ↓ [m]	PSNR ↑ [dB]	SSIM ↑	LPIPS ↓
None	Original mask	0.0624	30.95	0.886	0.121
Boundary variation	Erode 5 px	0.0643	30.56	0.883	0.117
	Erode 10 px	0.0649	30.25	0.881	0.126
	Dilate 5 px	0.0614	30.27	0.884	0.120
	Dilate 10 px	0.0616	30.84	0.887	0.118
Spatial shift	Horizontal ±5 px	0.0673	30.51	0.885	0.121
	Vertical ±5 px	0.0654	30.43	0.884	0.115
	Horizontal ±10 px	0.0700	30.40	0.885	0.124
	Vertical ±10 px	0.0638	30.83	0.891	0.115

**Table 6:** **Robustness to synthetic camera–sonar yaw perturbations.** For each nonzero perturbation magnitude, the results obtained with positive and negative yaw offsets are averaged. ↑: higher is better; ↓: lower is better.

Yaw Offset Magnitude	ATE RMSE ↓ [m]	PSNR ↑ [dB]	SSIM ↑	LPIPS ↓
$0^{\circ }$	0.0624	30.95	0.886	0.121
$2^{\circ }$	0.0631	30.46	0.883	0.120
$5^{\circ }$	0.0681	30.60	0.883	0.120
$10^{\circ }$	0.0665	30.66	0.883	0.122

**Table 7:** **Sensitivity analysis of key parameters.** The first row serves as the common default point for all three one-factor-at-a-time parameter sweeps, using a mask-loss weight of 0.1, a sonar intensity threshold of 0.15, and an association beam window of 0. Each remaining row varies one parameter while keeping all others fixed.↑: higher is better; ↓: lower is better.

Parameter	Value	ATE RMSE ↓ [m]	PSNR ↑ [dB]	SSIM ↑	LPIPS ↓
**Main setting**		0.0624	30.95	0.886	0.121
Mask-loss weight	0.025	0.0623	30.34	0.887	0.115
	0.050	0.0639	30.87	0.886	0.122
	0.200	0.0697	30.70	0.887	0.130
Sonar intensity threshold	0.09	0.0714	30.80	0.886	0.119
	0.21	0.0637	30.77	0.886	0.122
	0.30	0.0639	30.40	0.884	0.123
Association beam window	1	0.0644	30.70	0.883	0.124
	3	0.0648	30.67	0.888	0.115
	5	0.0626	30.53	0.883	0.129

**Table 8:** **Ablation study.** Performance comparison of different configurations on the 100 lb bomb sequence. The full method achieves the best tracking and rendering performance, demonstrating the effectiveness of sonar-guided depth-scale correction and object-aware mask supervision. ↑: higher is better; ↓: lower is better. The best results are shown in bold and denoted by first.

Configuration	ATE RMSE ↓ [m]	PSNR ↑	SSIM ↑	LPIPS ↓
(a) w/o Depth Scale	0.0642	29.45	0.838	0.213
(b) w/o Mask Loss	0.0632	30.28	0.884	0.123
Ours	**0.0624**	**30.95**	**0.886**	**0.121**

## Data Availability

The data used in this study are publicly available third-party datasets. The sources and links to these datasets are cited within the article.

## Abbreviations

The following abbreviations are used in this manuscript:

**Abbreviation**	**Definition**
3DGS	3D Gaussian Splatting
ATE	Absolute Trajectory Error
AUV	Autonomous Underwater Vehicle
CFAR	Constant False-Alarm Rate
ConvGRU	Convolutional Gated Recurrent Unit
DBA	Dense Bundle Adjustment
DVL	Doppler Velocity Log

FLS	Forward-Looking Sonar
GT	Ground Truth
iLBL–USBL	Hybrid Long/Inverted Ultra-Short Baseline
IMU	Inertial Measurement Unit
LPIPS	Learned Perceptual Image Patch Similarity
PSNR	Peak Signal-to-Noise Ratio
RGB	Red, Green, and Blue
RGB-D	Red, Green, Blue, and Depth
RMSE	Root Mean Square Error
ROV	Remotely Operated Vehicle
SfM	Structure from Motion
SLAM	Simultaneous Localization and Mapping
SSIM	Structural Similarity Index Measure
UXO	Unexploded Ordnance
